# Accelerated Phenotypical Aging in Midlife is Associated With Long-Term Cognitive Decline

**DOI:** 10.1093/geroni/igaf122.212

**Published:** 2025-12-31

**Authors:** Natascha Merten, Mary Ryan Baumann, Richard Chappell, Yanjun Chen, Lindsay Clark, Sterling Johnson, James Pankow, Art Walaszek

**Affiliations:** University of Wisconsin-Madison, Madison, Wisconsin, United States; University of Wisconsin-Madison, Madison, Wisconsin, United States; University of Wisconsin-Madison, Madison, Wisconsin, United States; University of Wisconsin-Madison, Madison, Wisconsin, United States; University of Wisconsin-Madison, Madison, Wisconsin, United States; University of Wisconsin-Madison, Madison, Wisconsin, United States; University of Minnesota, Minneapolis, Minnesota, United States; University of Wisconsin-Madison, Madison, Wisconsin, United States

## Abstract

PhenoAge, a multi-system biomarker uses easily-obtained common clinical blood tests and determines whether a person is younger or older on a biological and physiological level than expected by chronological age. Higher PhenoAge is associated with increased risk of disability, age-related morbidities and all-cause mortality. Its associations with early cognitive changes in midlife are less understood. The aim of this study was to determine whether accelerated PhenoAge in midlife was associated with 10-year cognitive changes in middle-aged to older adults. This longitudinal study is based on N = 2,630 (54% women;mean age 50years) Beaver Dam Offspring Study participants. We measured baseline blood-based clinical markers necessary for calculation of accelerated PhenoAge (PhenoAgeAccel). We tested Trail-making Test B (TMT-B) performance at baseline, 5-year and 10-year follow-up. We used linear mixed-effects model with PhenoAgeAccel as predictor and TMT-B time as outcome, adjusting for random intercepts, education and sex. Models were repeated sex-stratified. With every additional year older in PhenoAge compared to chronological age at baseline, participants performed worse on the TMT-B at baseline [overall:0.60 seconds slower, 95% Confidence Interval (0.29,0.91); women: 0.34(-0.05,0.72); men: 0.91(0.41,1.42)] and had a faster decline in TMT-B over 10-years [overall:0.94 seconds slower (0.58,1.29); women: 0.51(0.08,0.95); men: 1.48(0.90,2.07)]. Accelerated PhenoAge in midlife was associated with 10-year cognitive decline, overall and in men. Longer follow-up will be needed to determine whether PhenoAge might be predictive of cognitive impairment later in life. If confirmed, PhenoAge could become a cost-effective marker of cognitive decline and dementia and help identify at-risk individuals early and inform targeted interventions.

